# Detecting infection hotspots: Modeling the surveillance challenge for elimination of lymphatic filariasis

**DOI:** 10.1371/journal.pntd.0005610

**Published:** 2017-05-19

**Authors:** Julie R. Harris, Ryan E. Wiegand

**Affiliations:** 1 Division of Global Health Protection, Centers for Disease Control and Prevention, Atlanta, Georgia, United States of America; 2 Division of Parasitic Diseases and Malaria, Centers for Disease Control and Prevention, Atlanta, Georgia, United States of America; Imperial College London, Faculty of Medicine, School of Public Health, UNITED KINGDOM

## Abstract

**Background:**

During the past 20 years, enormous efforts have been expended globally to eliminate lymphatic filariasis (LF) through mass drug administration (MDA). However, small endemic foci (microfoci) of LF may threaten the presumed inevitable decline of infections after MDA cessation. We conducted microsimulation modeling to assess the ability of different types of surveillance to identify microfoci in these settings.

**Methods:**

Five or ten microfoci of radius 1, 2, or 3 km with infection marker prevalence (intensity) of 3, 6, or 10 times background prevalence were placed in spatial simulations, run in R Version 3.2. Diagnostic tests included microfilaremia, immunochromatographic test (ICT), and Wb123 ELISA. Population size was fixed at 360,000 in a 60 x 60 km area; demographics were based on literature for Sub-Saharan African populations. Background ICT prevalence in 6–7 year olds was anchored at 1.0%, and the prevalence in the remaining population was adjusted by age. Adults≥18 years, women aged 15–40 years (WCBA), children aged 6–7 years, or children≤5 years were sampled. Cluster (CS), simple random sampling (SRS), and TAS-like sampling were simulated, with follow-up testing of the nearest 20, 100, or 500 persons around each infection-marker-positive person. A threshold number of positive persons in follow-up testing indicated a suspected microfocus. Suspected microfoci identified during surveillance and actual microfoci in the simulation were compared to obtain a predictive value positive (PVP). Each parameter set was referred to as a protocol. Protocols were scored by efficiency, defined as the most microfoci identified, the fewest persons requiring primary and follow-up testing, and the highest PVP. Negative binomial regression was used to estimate aggregate effects of different variables on efficiency metrics.

**Results:**

All variables were significantly associated with efficiency metrics. Additional follow-up tests beyond 20 did not greatly increase the number of microfoci detected, but significantly negatively impacted efficiency. Of 3,402 protocols evaluated, 384 (11.3%) identified all five microfoci (PVP 3.4–100.0%) and required testing 0.73–35.6% of the population. All used SRS and 378 (98.4%) only identified all five microfoci if they were 2–3 km diameter or high-intensity (6x or 10x); 374 (97.4%) required ICT or Wb123 testing to identify all five microfoci, and 281 (73.0%) required sampling adults or WCBA. The most efficient CS protocols identified two (40%) microfoci. After limiting to protocols with 1-km radius microfoci of 3x intensity (n = 378), eight identified all five microfoci; all used SRS and ICT and required testing 31.2–33.3% of the population. The most efficient CS and TAS-like protocols as well as those using microfilaremia testing identified only one (20%) microfocus when they were limited to 1-km radius and 3x intensity.

**Conclusion:**

In this model, SRS, ICT, and sampling of adults maximized microfocus detection efficiency. Follow-up sampling of more persons did not necessarily increase protocol efficiency. Current approaches towards surveillance, including TAS, may not detect small, low-intensity LF microfoci that could remain after cessation of MDA. The model provides many surveillance protocols that can be selected for optimal outcomes.

## Author summary

Accurately tracking the success of elimination programs becomes increasingly difficult as elimination nears. For some diseases, such as lymphatic filariasis, this is compounded by the absence of symptoms during the infectious period as well as the natural and unevenly-distributed presence of small residual foci of infection (microfoci). Microfoci, when they are present, pose a threat to long-term elimination because they may lead to recrudescence, but it is unclear which surveillance approaches provide sufficient confidence to confirm their absence on a large scale. While we do not know the concentration of infections in a microfocus that would lead to certain recrudescence, we can quantitate the ability of different surveillance approaches to detect microfoci of different size and concentration. The model presented in this paper can be used to inform late-stage elimination program surveillance strategies for infections that include, but are not limited to, lymphatic filariasis.

## Introduction

Disease elimination is the endgame for much infectious disease-related public health work. Considered infinitely cost-effective when successful [1], elimination or eradication programs cost little per case prevented in the beginning, and enormous sums per case prevented at the end as efforts to prevent, detect, or treat every last case continue despite few remaining cases [2]. In part due to resource challenges and donor fatigue, efforts to eliminate infectious diseases have more often failed (malaria, yaws, yellow fever, hookworm) than succeeded (smallpox, rinderpest) [3]. Currently, several infectious diseases including polio, onchocerciasis, guinea worm, trachoma, malaria, and lymphatic filariasis are targeted for elimination or are at various stages of elimination or eradication programs [2, 4, 5].

Lymphatic filariasis (LF), a mosquito-borne filarial disease causing lymphedema, hydrocele, and elephantiasis has been targeted by the World Health Organization’s (WHO) Global Program to Eliminate Lymphatic Filariasis (GPELF) for elimination as a public health problem by 2020. For LF, this is defined as interruption of transmission using preventive chemotherapy, and management of morbidity and prevention of disability in persons already infected. The GPELF recommends steps to achieve interruption of transmission, including (i) mapping to define endemic areas; (ii) mass drug administration (MDA) in endemic areas to reduce infection below a threshold at which transmission is considered unsustainable; (iii) conducting and passing transmission assessment surveys (TAS) as a prerequisite for stopping MDA; (iv) post-treatment surveillance (PTS) after stopping MDA, comprising two repeat TAS and ongoing surveillance for at least five years; and (v) development of a dossier documenting these steps to achieve validation of the elimination of LF as a public health problem [6]. Although specifics of the last component are still to be determined, there is no doubt that complete elimination of LF transmission is the ultimate goal.

Unlike the elimination of smallpox or polio, the path to elimination for LF likely does not require the absence of every infection. This is largely due to the poor transmission characteristics of LF: multiple infective mosquito bites are needed to establish a patent infection with the causative filarial agents, and at least one pair of opposite-sex worms must be present for an infected person to manifest infectious microfilariae. The likelihood of both occurrences decreases as infection prevalence declines during multiple years of MDA [7–9]. Passing the TAS requires the identification of fewer infections during the survey than a pre-set cutoff, intended to signify a mean LF prevalence below which infections are likely to irreversibly decline. The TAS involves a community-based survey of 6–7 year old children in areas where school enrollment is <75%, or a school-based survey where enrollment is at least 75%. The design is usually a cluster survey, and the threshold is set at either <2% antigenemia (in *W*. *bancrofti*-endemic areas with *Anopheles* or *Culex* as the principal vector) or <1% antigenemia (in *W*. *bancrofti*-endemic areas where *Aedes* is the primary vector). In *Brugia*–endemic areas, thresholds are set for <2% antibody prevalence [10, 11]. ‘Passing’ the TAS—detecting no more positive children than the critical cutoff value specified in the guidelines—is a prerequisite for stopping MDA.

However, whether or not this cutoff universally leads to a decline in infections is unclear. The existence of LF—and other diseases, such as malaria—in endemic foci as small as 1 km in diameter [12–18] before or during MDA suggests at least the possibility of residual endemic foci after treatment. The few data that exist about LF in post-MDA settings suggest that there are residual foci [19–21] amid large areas relatively free of infection. In addition, the area and population over which TAS are carried out vary widely [22], and may include as many as 2 million persons. Thus, an average antigenemia prevalence of 1% or 2% among 6–7 year old children might look quite different in different areas, depending on multiple factors both affected and not affected by LF elimination program activities. Beyond this, the absence of infection markers in children is not necessarily associated with the absence of infection and transmission among adults. Even in post-MDA settings, adults have a higher prevalence of infection markers than children [19, 23–25]; whether or not these adults are actively transmitting infection is unclear. While single individuals infected with LF who are surrounded by large areas without infections are unlikely to restart active transmission cycles, there clearly exists a number and concentration of infected persons above which transmission will be sustained or expand. In these situations, the average antigenemia prevalence may indeed be below the cutoff in children aged 6–7 years without cessation of transmission; elimination of LF ‘as a public health problem’ may be briefly achieved, only to be lost in coming decades due to recrudescence.

For countries stopping MDA, PTS represents the last opportunity to detect any remaining foci of infection that may still lead to recrudescence [6, 10]. However, methods which efficiently detect and address infections—including small residual foci of infections—in a low-prevalence setting are undefined, particularly for a disease such as LF for which infectiousness and clinical symptoms may be separated by years or even decades [26–28]. In this paper, we use microsimulation modeling to compare the effectiveness of different programmatic surveillance approaches in detecting both residual endemic foci (‘microfoci’) and individual, dispersed infections. Data in this paper are intended to provide a realistic framework in which to consider surveillance for low-prevalence infections, not only for LF but also for other diseases targeted for elimination, and shed light on how much assurance different approaches to surveillance can provide during the last stages of an elimination program.

## Methods

### Model flow

**Fig 1:**
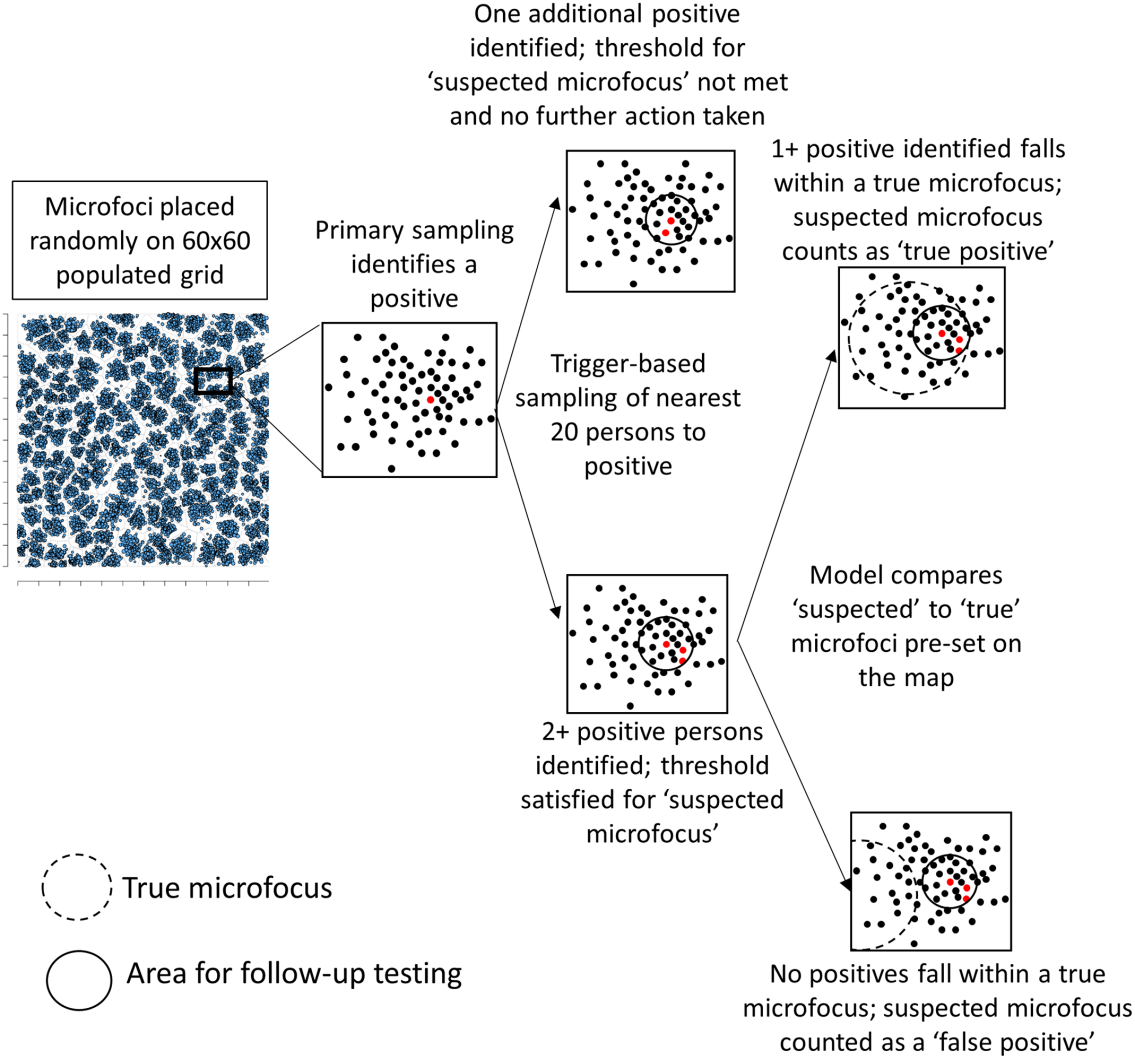
Example of simulation model. An area of the total population on which five microfoci are place is shown in an expanded view to describe the process when a person tests infection marker-positive. In this example, the trigger is one, trigger-based follow-up number is 20, and the threshold for action is two additional positives. An example of how the predictive value positive for identification of microfoci is calculated is shown on the far right, where persons testing positive during follow-up sampling either do or do not fall within an actual microfocus.

Microfoci, or areas of elevated infection prevalence relative to the background, were placed randomly on a map at the start of a simulation, with one household serving as the geographic center. Primary sampling included either 30-cluster sampling (CS) or simple random sampling (SRS), and only occurred in the population group specified by the simulation. The identification of a single infection-marker-positive person triggered follow-up testing (‘trigger-based sampling’) of the nearest *X* persons, irrespective of population group, around the initial positive (Fig 1, Table 1). Persons tested in trigger-based sampling included household members of the initial positive and persons living in the next-nearest households. A pre-set number of positives found during trigger-based sampling (‘threshold’) indicated the identification of a suspected microfocus (and the presumed requirement for action on the part of a country program). The number of true (known) microfoci was divided by the number of suspected microfoci to determine the predictive value positive of each simulation in identifying microfoci.

**Table 1:** Model parameters for sampling and identification of a microfocus.

Test steps	Test type		
	ICT	Microfilaria (mf)	Wb123
**Triggers for additional sampling during primary surveillance**	Any ICT-positive person	Any mf-positive person	Any Wb123-positive child ≤5 years of age
**Trigger-based sampling**	20, 100, 500 nearest persons (any age)	20, 100, 500 nearest persons (any age)	20, 100, 500 nearest persons (any age)
**Program manager believes he has identified a microfocus if he identifies during trigger-based sampling (*threshold*)**:	1, 2, 3, or 4 (trigger = 20) or 2, 4, 6, 8 (trigger = 100 or 500) additional ICT+ persons	1, 2, 3, or 4 additional mf-positive persons	40%, 50%, or 60% of persons tested are Wb123+, regardless of absolute number tested
**Actual identification of a microfocus (true positive) achieved if**:	Program manager believes he has identified a microfocus, **AND** Any positive person identified during follow-up resides within one of the ‘true’ microfoci on the map		

### Variables

**Fig 2:**
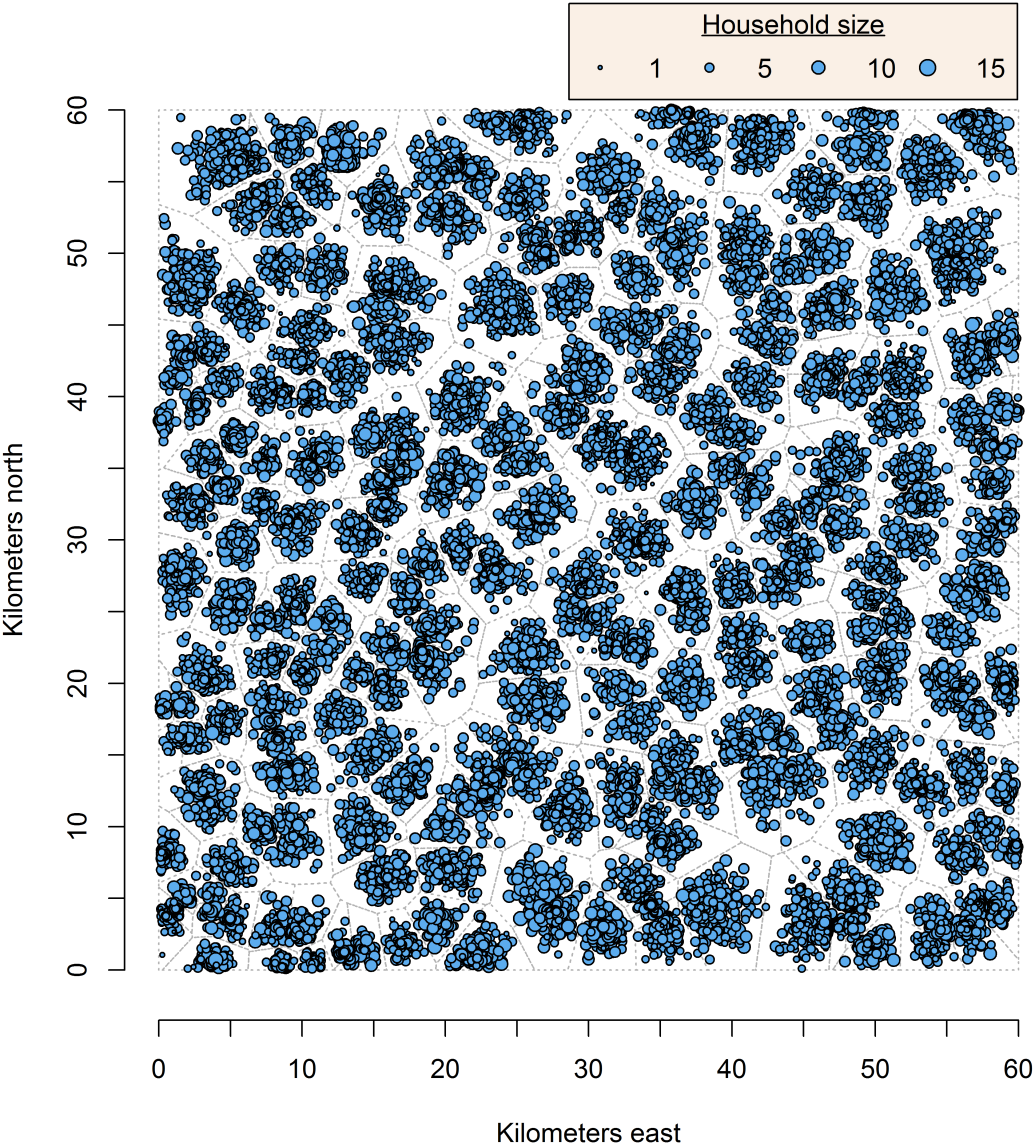
Example of simulation area. In a 60 x 60 km area, 300 villages are randomly distributed, with increasing household density towards a single, randomly-selected anchor household in each village. Size of the blue dot represents household size.

Each simulation utilized a 60 x 60 km region (3,600 km^2^), based on the approximate sizes of TAS evaluation areas in Chu *et al* [22], and included 360,000 persons with a mean population density of 100 persons/km^2^, similar to population densities of Ghana and Kenya [29]. The mean village size was 1,200 individuals; thus, each simulation comprised 300 villages. A mean household size of six was estimated from the literature [30] and World Family Map data from Ethiopia and Nigeria [31]; the simulation population was developed using population projections for Sub-Saharan Africa 2015 [32]. Villages were heterogeneously distributed throughout the area; a single household was chosen in each village as an ‘anchor’ and household density in each village decreased as distance from the anchor household increased (Fig 2). Due to the computation time required to create an area, ten simulation areas were created to use in modeling. Density plots of these areas and further details on how the ten areas were used in the simulation models are included in the supplementary materials (S1 Fig). The population proportion for children in 1-year age groups for children ≤5 years and 6–10 years was determined by dividing the total population proportion assigned to those age groups by five (Table 2). Background age-prevalence curves for infection markers were estimated based on the literature [19, 24, 25], using a background ICT prevalence among 6-7-year-old children of 1.0% to approximate a plausible post-MDA setting (Table 2).

**Table 2:** Population proportion and infection marker test status in background population. Microfilaremia was estimated to be 10-fold lower than ICT in every age group. Wb123 prevalence was assumed to be four-fold higher than ICT prevalence among persons <20 years of age, 4.5 times ICT prevalence among persons 20–40 years of age, and 5-fold greater than ICT prevalence among persons >40 years of age [19, 23–25]. Because these are based on test prevalences derived from real data (rather than gold standard prevalences for each infection marker), test sensitivity and specificity were not employed.

Ages (years)	Population %	ICT	microfilaremia	Wb123
0–1	3.24%	0.00%	0.00%	0.00%
>1–2	3.24%	0.17%	0.017%	0.67%
>2–3	3.24%	0.33%	0.033%	1.33%
>3–4	3.24%	0.50%	0.050%	2.00%
>4–5	3.24%	0.67%	0.067%	2.67%
>5–6	2.84%	0.83%	0.083%	3.33%
>6–7	2.84%	1.00%	0.100%	4.00%
>7–8	2.84%	1.00%	0.100%	4.00%
>8–9	2.84%	1.50%	0.150%	6.00%
>9–10	2.84%	1.75%	0.175%	7.00%
>10–20	24.00%	2.75%	0.275%	11.00%
>20–30	16.90%	3.75%	0.375%	16.88%
>30–40	12.00%	4.25%	0.425%	19.13%
>40–50	7.60%	4.25%	0.425%	21.25%
>50	9.70%	4.25%	0.425%	21.25%
**POP MEAN**	N/A	**2.75%**	**0.27%**	**12.30%**

Parameters that were varied in simulations included those related to the simulation area and those related to the sampling approach. Variables related to the simulation area included microfocus size (1, 2, or 3 km in radius), microfocus intensity (3x, 6x, or 10x greater prevalence of infection markers in the microfocus compared to the background infection marker prevalence), and the number of microfoci (5 or 10) included in each simulation, resulting in 18 different area types initially considered in the model. Variables related to the sampling and testing approach included test types used [microfilaremia blood smears, the immunochromatographic test (ICT) for filarial antigenemia, and Wb123 L3 larval antibody ELISA], population sampled [all adults ≥18 years of age (‘adults’), women of child-bearing age (ages 15–40) (‘WCBA’), children aged 6–7 years (‘6–7 yo’), or children aged ≤5 years (‘≤5 yrs’)], primary sampling methodology (CS or SRS), proportion of the population targeted during primary sampling [0.5% (1,800 persons sampled) or 2.0% (7,200 persons sampled) of the total population], number of persons required for trigger-based sampling (20, 100, or 500), and threshold number of positives (1, 2, 3, 4, 6, or 8) that indicated a required action, such as resumption of MDA (Table 2).

Primary Wb123 sampling only evaluated children aged ≤5 years, due to the potential for high prevalence of Wb123 antibodies among older age groups. The number of persons sampled in each cluster during CS was determined by the number of persons targeted for sampling, divided by 30 clusters. If the required sample size (1,800 or 7,200) for primary sampling was not reached in 30 clusters, all persons in the target age group in the 30 clusters were sampled. Additionally, a cluster sample comprising 1,548 6-7-year-olds was drawn to approximate a TAS.

Detailed definitions of the model terminology and a full list of outputs are included in the supplementary materials. All simulations were run in R versions 3.1 and 3.2 [33] (code available in S1 File).

Each set of simulated surveillance activities was termed a ‘protocol.’ We considered the priority of each protocol to maximize ‘efficiency’, defined as (in this order) maximizing the proportion of microfoci identified (which can also be thought of as the probability that any one microfocus is detected in each simulation), minimizing the number of total tests required to identify them, and maximizing the predictive value positive (PVP) of identification of microfoci. Protocols were sorted to optimize those three relevant outputs, with results reported as median proportions with 95% confidence intervals. The true proportion positive in each microfocus was tested against the background proportion positive for the targeted age group and test method to determine if a significantly higher proportion of positive persons was present in each microfocus. All comparisons were made with a two-sided Fisher’s Exact test. The number of statistically significant associations was summed for each protocol and the percentage of microfoci with statistically more infections than background prevalence was recorded. Negative binomial regression was used to estimate aggregate differences across all protocols in regression analyses. Results of regression analyses are reported as rate ratios with 95% confidence intervals and p-values.

## Results

### Regression analyses

In total, 6,804 protocols were identified and evaluated in regression analyses, presented in Tables 3 and 4. Modifying nearly all variables included in the model significantly affected the three relevant outputs.

**Table 3:** Effect of varying the model variables on the median predictive value positive of identifying microfoci, the median proportion of the population requiring testing, and the median proportion of microfoci detected. In this analysis, TAS-like protocols are excluded. The threshold for all microfilaremia testing is set at 1. For ICT tests, the threshold for identification of a suspected microfocus is 1 positive when 20 follow-up tests are used, and 2 in all other cases. For Wb123 testing, the threshold is 50% of the persons followed up testing positive.

	Predictive value positive			Proportion population tested		Proportion microfoci detected	
Variable	Level	RR (CI)	p	RR (CI)	P	RR (CI)	P
**Number of microfoci**	5	ref		Ref		ref	
	10	1.69 (1.57–1.84)	< 0.001	1.05 (1.00–1.10)	0.03	1.04 (0.98–1.09)	0.19
**Microfocus radius**	1 km	ref		Ref		ref	
	2 km	1.52 (1.37–1.69)	< 0.001	1.03 (0.98–1.09)	0.26	1.42 (1.32–1.52)	< 0.001
	3 km	1.85 (1.67–2.05)	< 0.001	1.07 (1.017–1.13)	0.010	1.74 (1.63–1.86)	< 0.001
**Intensity**	3x	ref		Ref		ref	
	6x	2.86 (2.54–3.24)	< 0.001	1.04 (0.98–1.10)	0.17	1.62 (1.51–1.75)	< 0.001
	10x	3.65 (3.24–4.12)	< 0.001	1.08 (1.02–1.14)	0.008	1.91 (1.78–2.05)	< 0.001
**Sampling proportion**	0.005	ref		Ref		ref	
	0.02	1.01 (0.94–1.09)	0.74	2.95 (2.81–3.11)	< 0.001	1.52 (1.44–1.61)	< 0.001
**Sampling method**	CS	ref		Ref		ref	
	SRS	1.38 (1.27–1.49)	< 0.001	1.95 (1.86–2.04)	< 0.001	4.05 (3.79–4.34)	< 0.001
**Test method**	ICT	ref		Ref		ref	
	mf	1.82 (1.64–2.03)	< 0.001	0.31 (0.29–0.32)	< 0.001	0.28 (0.26–0.30)	< 0.001
	Wb	6.38 (5.75–7.08)	< 0.001	0.76 (0.72–0.81)	< 0.001	0.67 (0.62–0.72)	< 0.001
**Follow-up tests**	20	ref		Ref		ref	
	100	0.90 (0.82–0.99)	0.03	1.90 (1.76–2.05)	< 0.001	1.15 (1.08–1.23)	< 0.001
	500	0.77 (0.70–0.85)	< 0.001	4.93 (4.61–5.27)	< 0.001	1.21 (1.14–1.30)	< 0.001

**Table 4:** Effect of varying the model variables on the median predictive value positive of identifying microfoci, the median proportion of the population requiring testing, and the median proportion of microfoci detected. In this analysis, both TAS-like protocols and Wb123 protocols are excluded. The threshold for all microfilaremia testing is set at 1. For ICT tests, the threshold for identification of a suspected microfocus is 1 positive when 20 follow-up tests are used, and 2 in all other cases.

	Predictive value positive			Proportion population tested		Proportion microfoci detected	
Variable	Level	RR (CI)	p	RR (CI)	p	RR (CI)	P
**Number of microfoci**	5	ref		Ref		ref	
	10	2.12 (1.94–2.31)	< 0.001	1.05 (1.01–1.08)	0.006	1.03 (0.98–1.09)	0.18
**Microfocus radius**	1 km	ref		Ref		ref	
	2 km	1.87 (1.66–2.11)	< 0.001	1.03 (0.99–1.08)	0.15	1.42 (1.33–1.51)	< 0.001
	3 km	2.73 (2.45–3.06)	< 0.001	1.07 (1.03–1.12)	0.001	1.76 (1.65–1.87)	< 0.001
**Intensity**	3x	ref		Ref		ref	
	6x	2.04 (1.81–2.32)	< 0.001	1.04 (1.00–1.08)	0.08	1.35 (1.26–1.44)	< 0.001
	10x	3.24 (2.88–3.64)	< 0.001	1.07 (1.03–1.12)	< 0.001	1.61 (1.51–1.71)	< 0.001
**Sampling proportion**	0.005	ref		ref		ref	
	0.02	0.98 (0.91–1.07)	0.67	2.92 (2.81–3.03)	< 0.001	1.55 (1.47–1.63)	< 0.001
**Sampling method**	CS	ref		ref		ref	
	SRS	1.73 (1.59–1.88)	< 0.001	2.01 (1.94–2.08)	< 0.001	3.92 (3.68–4.17)	<0.001
**Test method**	ICT	ref		ref		ref	
	mf	1.82 (1.68–1.99)	< 0.001	0.31 (0.29–0.32)	< 0.001	0.28 (0.26–0.30)	< 0.001
**Follow-up tests**	20	ref		ref		ref	
	100	1.00 (0.91–1.10)	0.97	1.89 (1.79–2.01)	< 0.001	1.21 (1.13–1.28)	< 0.001
	500	0.73 (0.66–0.81)	< 0.001	4.85 (4.61–5.11)	< 0.001	1.29 (1.21–1.37)	< 0.001

Maximizing the PVP is critical to protocol efficiency; protocols with low PVP waste resources by causing follow-up actions on suspected microfoci that are not truly microfoci. Increasing the number of microfoci primarily affected the PVP: having more microfoci in the model increased the likelihood that any microfocus investigated would be a ‘true’ microfocus. Similarly, increasing the radius of each microfocus increased the PVP, but had less effect on the median proportion of persons tested in each protocol. However, it did significantly increase the median proportion of microfoci detected.

Increasing the intensity of the microfocus caused a large and significant increase in PVP and the proportion of microfoci detected in each model, but had a smaller effect on the proportion of persons tested. Increasing the proportion of the population sampled in primary sampling increased the median total proportion of persons tested, but also increased the median proportion of microfoci detected, without affecting PVP (that is, more true microfoci were detected with increased primary sampling, but the number of false positive microfoci did not increase). All three metrics were significantly improved by using SRS instead of CS as the primary sampling methodology. In contrast, when using Wb123 or microfilaremia testing, compared with ICT, only PVP was increased; the proportion of persons tested declined but the median proportion of microfoci detected also declined significantly.

Increasing the number of follow-up tests generally caused a decrease in the PVP and had relatively small—though significant—effects on the proportion of microfoci detected. However, it did have a large and significant effect on the proportion of persons requiring testing (Table 3), with nearly five times more persons requiring testing when 500 persons were followed-up instead of 20 persons.

To investigate the effect of testing different age groups on the same metrics, we additionally excluded Wb123 testing, which only included children ≤5 years of age. The results for all other variables (Table 4) were largely the same as described above (Table 3). Results for women of child-bearing age are largely the same as results for all adults: compared with testing children, testing adults generally increases the PVP minimally, and increases both the proportion of the population tested and the proportion of microfoci identified more substantially.

Based on the above results and to represent likely post-MDA scenarios as simply as possible, for the remaining analyses we limited the number of microfoci to five (n = 3,402 protocols). All 3,402 protocols with relevant inputs and outputs are available in S1 Table.

### Description of microfoci

Microfoci are described in Table 5. Although most simulations resulted in more infections in each microfocus compared with expected infections at background prevalence, we separated the simulations into those where >80% vs ≤80% of microfoci had statistically significantly more infections than would be expected (Table 5, shaded vs unshaded boxes). In total, 57 (70%) of the 81 simulation combinations shown had >80% of microfoci with statistically significantly more positives, as measured by the target infection marker in the target age group, than expected at background rates. Simulations with larger and more intense microfoci, those utilizing more sensitive tests, and those involving adults were more likely than others to have statistically significantly more infections in each microfoci than background.

**Table 5:** Characteristics of microfoci. Shaded boxes represent those with <80% of microfoci in each simulation being statistically significantly different from background, in terms of infection prevalence. Test: test type. Rad: Microfocus radius. Int: Microfocus intensity. Med pop size: median target population size in microfocus. Med pos: Median number of positives by test type in microfocus. Expected pos: Expected number of positives by test type in microfocus at background prevalence. Prop microfoci p<0.05: Proportion of microfoci across all simulations in that category with more positive persons (p<0.05) in target age group than background.

Characteristic			Children <5				6–7 yo				Adults				WCBA			
Test	Rad	Int	Med pop size	Med pos	Expected pos	Prop microfoci p<0.05	Med pop size	Med pos	Expected pos	Prop microfoci p<0.05	Med pop size	Med pos	Expected pos	Prop microfoci p<0.05	Med pop size	Med pos	Expected pos	Prop microfoci p<0.05
ICT	1 km	3x	215	3	0.88	20.1%	64	2	0.64	10.6%	567	67	22.43	98.7%	226	25	8.24	93.7%
ICT	2 km	3x	379	4	1.55	57.5%	113	4	1.13	44.0%	1000	118.5	39.56	99.9%	400	44	14.59	99.5%
ICT	3 km	3x	596	7	2.43	91.9%	178	6	1.78	79.2%	1582	188	62.56	100.0%	632	69	23.05	100.0%
ICT	1 km	6x	217	5	0.89	86.5%	64	4	0.64	82.0%	571	135	22.59	99.9%	228	50	8.31	98.8%
ICT	2 km	6x	377	10	1.54	99.0%	113	7	1.13	98.6%	1000	237.5	39.56	100.0%	400	87	14.59	100.0%
ICT	3 km	6x	597	14	2.44	99.9%	178	11	1.78	100.0%	1582	376	62.58	100.0%	633	138	23.08	100.0%
ICT	1 km	10x	216	9	0.88	92.5%	64	6	0.64	92.8%	572	226	22.63	100.0%	227	83	8.28	99.6%
ICT	2 km	10x	378	15	1.54	99.5%	113	11	1.13	99.5%	1005	398	39.76	100.0%	401	147	14.62	100.0%
ICT	3 km	10x	593	24	2.42	100.0%	178	18	1.78	100.0%	1566	619	61.95	100.0%	626	229	22.83	100.0%
MF	1 km	3x	215	0	0.09	0.0%	64	0	0.06	0.0%	567	7	2.24	80.8%	226	2	0.82	32.8%
MF	2 km	3x	379	0	0.15	0.0%	113	0	0.11	0.0%	1000	12	3.96	98.3%	400	4	1.46	55.4%
MF	3 km	3x	596	0	0.24	0.0%	178	0	0.18	0.1%	1582	19	6.26	99.9%	632	7	2.30	73.8%
MF	1 km	6x	217	0	0.09	0.1%	64	0	0.06	0.5%	571	13	2.26	94.7%	228	5	0.83	80.1%
MF	2 km	6x	377	0	0.15	2.0%	113	0	0.11	9.8%	1000	24	3.96	99.8%	400	9	1.46	95.9%
MF	3 km	6x	597	0	0.24	13.9%	178	2	0.18	49.1%	1582	38	6.26	100.0%	633	14	2.31	99.5%
MF	1 km	10x	216	0	0.09	4.2%	64	0	0.06	11.2%	572	23	2.26	97.2%	227	8	0.83	91.9%
MF	2 km	10x	378	0	0.15	29.8%	113	2	0.11	54.5%	1005	40	3.98	100.0%	401	15	1.46	99.5%
MF	3 km	10x	593	3	0.24	82.5%	178	2	0.18	94.4%	1566	62	6.2	100.0%	626	23	2.28	100.0%
WB	1 km	3x	215	11	3.51	87.2%	64	8		84.2%	567	313		100.0%	226	109		99.2%
WB	2 km	3x	379	18	6.18	99.0%	113	14		98.7%	1000	552		100.0%	400	193		99.9%
WB	3 km	3x	596	29	9.72	99.9%	178	21		99.9%	1582	873		100.0%	632	305		100.0%
WB	1 km	6x	217	21	3.54	95.7%	64	15		95.7%	571	553.5		100.0%	228	208		99.9%
WB	2 km	6x	377	37	6.15	99.8%	113	27		99.9%	1000	971		100.0%	400	364		100.0%
WB	3 km	6x	597	58	9.74	100.0%	178	43		100.0%	1582	1537		100.0%	633	576		100.0%
WB	1 km	10x	216	35	3.52	98.3%	64	26		97.9%	572	572		100.0%	227	227		99.9%
WB	2 km	10x	378	62	6.17	100.0%	113	45		100.0%	1005	1005		100.0%	401	401		100.0%
WB	3 km	10x	593	97	9.68	100.0%	178	71		100.0%	1566	1566		100.0%	626	626		100.0%

### Protocols which identify the most microfoci

Of the 3,402 protocols, 384 (11.3%) identified all five microfoci. The number of protocols identifying all five microfoci declined as size and intensity of the microfoci decreased (Table 6).

**Table 6:** Number of protocols identifying 100% of the microfoci at different microfocus sizes and intensities.

Microfocus radius (km)	Microfocus intensity	Median microfocus sensitivity	Number of protocols available
3	10	100%	88
3	6	100%	66
3	3	100%	26
2	10	100%	69
2	6	100%	56
2	3	100%	18
1	10	100%	31
1	6	100%	22
1	3	100%	8
**TOTAL**	**—**	**—**	**384**

The 384 protocols required testing a range of 0.73%-35.6% of the total population, and had a range of PVPs of 3.2–100.0%. All used SRS and 378 (98.4%) only identified all five microfoci if they were 2–3 km diameter or high-intensity (6x or 10x) (Table 6). Of the 384, 374 (97.4%) required ICT or Wb123 testing to identify all five microfoci, and 281 (73.0%) required sampling adults or WCBA. The top 10 most efficient protocols (those which identified the most microfoci with the fewest tests at the highest PVP) are shown in Table 7.

**Table 7:** Inputs and outputs of the most efficient surveillance protocols. Test: Test type used. Samp: Sampling methodology. PP: Population proportion sampled. Ages: ages sampled during primary sampling. Radius: microfocus radius. Int: microfocus intensity. TBS: Number tested in trigger-based sampling (all ages). TH: Threshold (number of positives required during trigger-based sampling for program manager to believe they have identified a microfocus). Pos/Test: Proportion of persons tested who are positive. Pos/PopPos: Proportion of all positive persons in the population who are identified in testing. Test/Pop: Proportion of total population tested. **μf** PVP: Proportion of all suspected microfoci that are true microfoci (correctly identified as microfoci). **μf** Sens: Proportion of all microfoci found through the sampling protocol.

Test	Samp	PP	Ages	Radius	Int	TBS	TH^1^	Pos/Test	Pos/PopPos	μf PVP (median, 95% CI)	Test/Pop (median, 95% CI)	μf Sens (median, 95% CI)
WB	SRS	0.50%	<5 yrs	3	10	20	10	11.75%	0.56%	57.1% (37.5–83.3)	0.73% (0.66–0.80)	100% (60–100)
WB	SRS	0.50%	<5 yrs	3	10	20	12	11.75%	0.56%	57.1% (38.5–100.0)	0.73% (0.66–0.80)	100% (60–100)
WB	SRS	0.50%	<5 yrs	3	10	20	8	11.75%	0.56%	55.6% (36.4–83.3)	0.73% (0.66–0.80)	100% (60–100)
ICT	SRS	0.50%	WCBA	3	6	20	3	5.01%	1.40%	50.0% (33.3–83.3)	0.94% (0.85–1.04)	100% (60–100)
ICT	SRS	0.50%	WCBA	3	6	20	2	5.01%	1.40%	30.8% (21.1–45.5)	0.94% (0.85–1.04)	100% (60–100)
ICT	SRS	0.50%	WCBA	3	6	20	1	5.01%	1.40%	13.5% (9.7–19.2)	0.94% (0.85–1.04)	100% (80–100)
ICT	SRS	0.50%	Adults	2	6	20	1	4.66%	1.40%	12.9% (8.1–18.5)	0.95% (0.86–1.04)	100% (60–100)
ICT	SRS	0.50%	WCBA	2	10	20	4	6.35%	1.76%	66.7% (45.5–100.0)	0.96% (0.85–1.05)	100% (60–100)
ICT	SRS	0.50%	WCBA	2	10	20	3	6.35%	1.76%	56.4% (38.5–83.8)	0.96% (0.85–1.05)	100% (60–100)
ICT	SRS	0.50%	WCBA	2	10	20	2	6.35%	1.76%	33.3% (22.7–55.6)	0.96% (0.85–1.05)	100% (80–100)

^***1***^For Wb123 testing only, thresholds for identifying a suspected microfocus are set at 40%, 50%, or 60% of the persons tested in trigger-based sampling. In this table and in the following tables, thresholds are expressed as whole numbers (i.e., a threshold of 8 positives with a trigger-based sampling number of 20 corresponds to 40%, etc.).

### Protocols which identify small, low-intensity microfoci

The most efficient protocols identified during this initial evaluation invariably involved models with high-intensity (6x or 10x) and large (radius 2–3 km) microfoci. However, most residual microfoci post-MDA are likely to be low-intensity and small. To address this, we limited our model to include only protocols with microfoci of size 1 km in radius and 3x intensity. Of these, eight (2.1%) protocols identified all five microfoci; nine (2.4%) identified four; 13 (3.4%) identified three; 18 (4.8%) identified two; 39 (10.3%) identified one; 291 (77.0%) identified no microfoci. Among the eight identifying all five microfoci, each required testing of 31%-33% of the population. As the proportion of microfoci identified decreased, the proportion of the population requiring testing similarly decreased. In Table 8, we show a sample of the most efficient protocols when 100%, 80%, 60%, or 40% of microfoci are identified.

**Table 8:** Protocols which required the fewest total persons to be tested to identify 100%, 80%, 60%, or 40% of the microfoci of size 1 km in radius and 3x in intensity.

Test	Samp	PP	Ages	Radius	Int	TBS	TH	Pos/Test	Pos/PopPos	μf PVP (median, 95% CI)	Test/Pop (median, 95% CI)	μf Sens (median, 95% CI)
ICT	SRS	2.00%	WCBA	1	3	500	8	2.96%	32.70%	3.3% (2.0–3.9)	31.28% (28.54–34.26)	100% (60–100)
ICT	SRS	2.00%	WCBA	1	3	500	6	2.96%	32.70%	3.2% (2.0–3.9)	31.28% (28.54–34.26)	100% (60–100)
ICT	SRS	2.00%	WCBA	1	3	500	2	2.96%	32.70%	3.2% (2.0–3.9)	31.28% (28.54–34.26)	100% (60–100)
ICT	SRS	2.00%	WCBA	1	3	500	4	2.96%	32.70%	3.2% (2.0–3.9)	31.28% (28.54–34.26)	100% (60–100)
ICT	SRS	2.00%	WCBA	1	3	20	1	3.42%	4.17%	4.3% (2.1–6.0)	3.46% (3.30–3.64)	80% (40–100)
ICT	SRS	2.00%	Adults	1	3	20	1	3.59%	4.55%	4.1% (2.0–5.6)	3.59% (3.43–3.77)	80% (40–100)
ICT	SRS	2.00%	WCBA	1	3	100	6	3.14%	10.06%	22.7% (11.8–36.4)	9.07% (8.34–9.89)	80% (40–100)
ICT	SRS	2.00%	WCBA	1	3	100	4	3.14%	10.06%	6.5% (3.5–8.6)	9.07% (8.34–9.89)	80% (40–100)
ICT	SRS	2.00%	WCBA	1	3	20	2	3.42%	4.17%	11.5% (3.8–20.8)	3.46% (3.30–3.64)	60% (20–100)
ICT	SRS	2.00%	Adults	1	3	20	2	3.59%	4.55%	11.1% (3.6–19.1)	3.59% (3.43–3.77)	60% (20–100)
ICT	SRS	2.00%	6–7 yo	1	3	100	4	2.02%	2.84%	11.8% (0.0–22.7)	3.99% (3.61–4.39)	60% (0–100)
ICT	SRS	2.00%	6–7 yo	1	3	100	2	2.02%	2.84%	5.5% (1.7–9.6)	3.99% (3.61–4.39)	60% (20–100)
ICT	SRS	0.50%	WCBA	1	3	20	1	3.44%	1.07%	6.9% (0.0–15.4)	0.88% (0.79–0.95)	40% (0–80)
ICT	SRS	0.50%	Adults	1	3	20	1	3.59%	1.15%	6.9% (0.0–13.8)	0.90% (0.81–1.00)	40% (0–80)
ICT	SRS	0.50%	WCBA	1	3	100	6	3.14%	2.63%	33.3% (0.0–100)	2.38% (1.94–2.74)	40% (0–80)
ICT	SRS	0.50%	WCBA	1	3	100	4	3.14%	2.63%	11.1% (0.0–23.5)	2.38% (1.94–2.74)	40% (0–80)

Because all of the ‘most efficient’ protocols in the preceding examples specified ICT and SRS, we explored the most efficient options using different test types and diagnostic methods with small, low-intensity microfoci. Table 9 shows the most efficient protocols using microfilaria testing, Wb123 testing, or cluster sampling. The most efficient protocols using CS, MF, or Wb123 fail to identify more than one or two of the five microfoci and are markedly less efficient than the ICT/SRS protocols shown in Table 8.

**Table 9:** The most efficient protocols using cluster sampling, microfilaria testing, and WB123 testing in settings with microfoci of size 1 km in radius and 3x in intensity.

Test	Samp	PP	Ages	Radius	Int	TBS	TH	Pos/Test	Pop/PopPos	μf PVP (median, 95% CI)	Test/Pop (median, 95% CI)	μf Sens (median, 95% CI)
ICT	**CS**	2.00%	Adults	1	3	20	1	3.50%	3.76%	2.5% (0.0–7.2)	3.05% (2.92–3.20)	20% (0–40)
**MF**	SRS	2.00%	WCBA	1	3	500	3	0.32%	6.29%	16.7% (0.0–66.7%)	5.54% (4.5–6.9%)	20% (0–60)
**WB**	SRS	2.00%	<5 yrs	1	3	20	8	4.66%	0.98%	100.0% (50–100)	2.66% (2.54–2.78)	40% (0–80)

### Children vs adults

Different settings will facilitate sampling of different populations with greater ease. For this reason, we compared the most efficient sampling protocols limited to 6–7 year olds, WCBA, and adults. To facilitate a fair comparison across population groups, we limited the radius of microfoci to 1 km, the intensity to 3x, and the threshold to 1. The results are shown in Table 10. Both WCBA and adult sampling outperform sampling of 6–7 year olds in terms of efficiency. Children <5 years were not included in this evaluation as they were only evaluated with Wb123.

**Table 10:** The most efficient protocols using microfilaria testing using a 1-km radius, 3x intensity, and threshold of 1 for 6–7 yo, WCBA, and adults>18 years.

Test	Samp	PP	Ages	Radius	Int	TBS	TH	Pos/Test	Pop/PopPos	μf PVP (median, 95% CI)	Test/Pop (median, 95% CI)	μf Sens (median, 95% CI)
ICT	SRS	2.00%	6–7 yo	1	3	20	1	1.36%	1.16%	7.1 (0.0–14.7)	2.40% (2.32–2.49)	40% (0–80)
ICT	SRS	2.00%	WCBA	1	3	20	1	3.42%	4.17%	4.3% (2.1–6.0)	3.46% (3.30–3.64)	80% (40–100)
ICT	SRS	0.50%	adults	1	3	20	1	3.59%	4.55%	4.1% (2.0–5.6)	3.59% (3.43–3.77)	80% (40–100)

### Cluster sampling protocols

Simple random sampling consistently resulted in higher efficiencies than cluster sampling. However, recognizing that simple random sampling might not always be feasible, we evaluated the peak efficiency of cluster sampling protocols at varying microfoci radii and intensities. In total, 1,458 protocols used cluster sampling (*not* including TAS-like sampling). The protocols identified 0%-40% of the five microfoci, and tested 0.9%-8.0% of the total population. Only 74 (5%) of the 1,458 protocols identified 40% of the microfoci; the remainder identified fewer. All involved large (3-km), high-intensity (10x) microfoci. The three most efficient protocols with cluster sampling are presented in Table 11.

**Table 11:** The most efficient protocols using cluster sampling methodology without limiting microfocus radius or intensity.

Test	Samp	PP	Ages	Radius	Int	TBS	TH	Pos/Test	Pop/PopPos	μf PVP (median, 95% CI)	Test/Pop (median, 95% CI)	μf Sens (median, 95% CI)
ICT	CS	0.50%	Adults	3	10	20	1	5.97%	1.35%	7.9% (0.0–20.0)	0.87% (0.76–1.07)	40% (0–80)
ICT	CS	0.50%	WCBA	3	10	20	1	6.20%	1.45%	8.3% (0.0–21.1)	0.90% (0.78–1.14)	40% (0–80)
ICT	CS	0.50%	WCBA	3	10	100	6	6.04%	3.58%	33.3% (0.0–100)	2.28% (1.84–2.99)	40% (0–80)

The targeted primary sample size was not achieved for 180 of 729 cluster sampling protocols specifying 2.0% primary sampling; all involved 6–7 year olds.

### Sampling protocols that use microfilaremia testing

Microfilaria testing is used in many countries despite challenges with sensitivity, which decreases as the prevalence of infection declines. As demonstrated above, for small, low-intensity microfoci, child populations are much less likely than adults or WCBA to have statistically more microfilaremia-positive persons than background (Table 5), and thus detection of any microfocus using microfilaremia testing in children is unlikely (or due to chance). In total, 1,458 protocols used microfilaria testing; of these, 10 identified 100% of the microfoci by testing 3.0%-7.0% of the population. These primarily involved large, high-intensity microfoci. When the parameters were limited to microfoci of radius 1 km and 3x intensity, the highest proportion of microfoci that could be identified was 20%, by testing 5.5%-5.9% of the population. Table 12 shows the top two most efficient protocols when 100%, 80%, 60%, or 40% of the microfoci are identified. Table 13 shows the three most efficient protocols using microfilaremia testing when microfoci are limited to radius 1 km and 3x intensity. Notably, even though 81% of the 1 km, 3x-intensity microfoci have statistically greater numbers of infected adults than background as indicated by microfilaremia testing (Table 5), the most efficient protocol in adults detects only one in five microfoci (Table 13).

**Table 12:** The most efficient protocols using microfilaria testing without limiting microfocus radius or intensity.

Test	Samp	PP	Ages	Radius	Int	TBS	TH	Pos/Test	Pop/PopPos	μf PVP (median, 95% CI)	Test/Pop (median, 95% CI)	μf Sens (median, 95% CI)
Mf	SRS	2.00%	WCBA	3	10	100	1	0.68%	5.26%	33.3% (21.4–50.1)	3.00% (2.71–3.32)	100% (60–100)
Mf	SRS	2.00%	Adults	3	10	100	1	0.71%	5.62%	33.3% (20.0–55.6)	3.07% (2.75–3.42)	100% (60–100)
Mf	SRS	2.00%	WCBA	2	10	100	1	0.57%	4.75%	35.7% (20.0–60.0)	2.90% (2.60–3.22)	80% (40–100)
Mf	SRS	2.00%	Adults	3	6	100	1	0.50%	4.39%	30.8 (14.3–55.6)	2.93% (2.65–3.25)	80% (20–100)
Mf	SRS	2.00%	WCBA	3	10	20	1	0.56%	3.17%	57.1% (25.0–100.0)	2.20% (2.14–2.27)	60% (20–100)
Mf	SRS	2.00%	Adults	3	10	20	1	0.60%	3.46%	57.1 (28.6–100.0)	2.22 (2.15–2.29)	60% (20–100)
Mf	SRS	0.50%	WCBA	3	10	100	2	0.70%	1.37%	100.0% (33.3–100.0)	0.75% (0.61–0.92)	40% (0–80)
Mf	SRS	0.50%	WCBA	3	10	100	1	0.70%	1.37%	50.0% (0.0–100.0)	0.75% (0.61–0.92)	40% (0–80)

**Table 13:** The most efficient protocols using microfilaremia testing when microfoci are limited to 1 km radius and 3x intensity.

Test	Samp	PP	Ages	Radius	Int	TBS	TH	Pos/Test	Pop/PopPos	μf PVP (median, 95% CI)	Test/Pop (median, 95% CI)	μf Sens (median, 95% CI)
Mf	SRS	2.00%	WCBA	1	3	500	1	0.32%	6.29%	5.6% (0.0–16.7)	5.5% (4.5–6.9)	20% (0–60)
Mf	SRS	2.00%	WCBA	1	3	500	1	0.33%	6.51%	11.1% (0.0–26.3)	5.7% (4.5–7.2)	20% (0–60)
Mf	SRS	2.00%	Adults	1	3	500	1	0.33%	6.88%	5.0% (0.0–15.8)	5.9% (4.6–7.3)	20% (0–60)

### TAS-like sampling

To evaluate how well the TAS would perform at detecting microfoci of various sizes, we simulated a TAS (as described in the Methods). As with the other protocols, each of these did incorporate trigger-based follow-up testing around each positive and a threshold for determining a suspected microfocus; thus, each demonstrates how few people could be tested and how many microfoci found *if* follow-up testing around infected persons was carried out.

There were 486 protocols that utilized TAS-like sampling. The most efficient of these, using each diagnostic tool, are shown in Table 14. Although each required testing <1% of the population, none of the protocols identified more than one of the five microfoci in each simulation.

**Table 14:** Efficiency of TAS-like sampling at detecting microfoci using ICT.

Test	Samp	Ages	Radius	Int	TBS	TH	Pos/Test	Pop/PopPos	μf PVP (median, 95% CI)	Test/Pop (median, 95% CI)	μf Sens (median, 95% CI)
ICT	TAS	6–7 yo	2	6	20	1	1.63%	0.27%	33.3% (0.0–100.0)	0.52% (0.48–0.57)	20% (0–40)
ICT	TAS	6–7 yo	2	6	20	1	1.70%	0.28%	33.3% (0.0–100.0)	0.52% (0.48–0.58)	20% (0–60)
ICT	TAS	6–7 yo	3	6	20	1	1.90%	0.30%	50.0% (0.0–100.0)	0.53% (0.48–0.59)	20% (0–60)

## Discussion

Endemic foci that lead to stable or increasing numbers of infections threaten the success of infectious disease elimination or eradication programs. Current approaches to post-treatment surveillance for LF require that we assume the absence of infections in between well-defined areas where infections are known to be absent. Due to the highly focal nature of LF endemicity, this approach may not be sufficient to confirm LF elimination. The model presented here provides several important pieces of information about what can be expected from various types of surveillance in terms of identifying small endemic disease foci.

First, we confirm the challenges in detecting small, low-intensity microfoci. While this in itself is unsurprising, this model demonstrates precisely how much additional effort is needed to identify microfoci—and which diagnostic test and follow-up testing combinations can do so most efficiently—as they become incrementally smaller and/or less intense. Programs may wish to have a specific level of confidence in their ability to detect microfoci of specific size, as measured by a specific marker: this model enables them to select approaches that could yield that level of certainty. While many protocols enabled the detection of all five large (3-km radius), high-intensity (10x) microfoci while testing small proportions of the total population, the only protocols that identified all five small, low-intensity microfoci required testing of an impractically high number of persons (>30% of the population). Notably, there are some protocols (Table 8) which identify most of the microfoci– 4 of 5 –and require testing of only 3.5% of the population (12,600 persons in this model). While this may seem like a high number, the use of protocols such as this two years in a row might provide reasonable confidence in the absence of microfoci. These protocols include simple random sampling of adults or women of childbearing age using ICT, conducting follow-up testing of the 20 nearest persons to any identified positives, and using a threshold of just one additional infected person to identify areas needing additional programmatic attention. One way to achieve this in a country with high antenatal clinic attendance might be to test all women attending antenatal clinics until the sample size is reached for two years in a row. Interestingly, follow-up sampling of 500 persons around each infected person in this model, as is done in Togo [34], did not appear to provide more confidence in detection of microfoci than follow-up sampling of 20 persons, and was carried out at a large cost to the predictive value positive.

The model also demonstrates that microfoci are exceedingly difficult to detect during PTS using a tool as insensitive as microfilaria smears. This due to the low estimated prevalence of microfilaremia in a post-MDA population, and the declining sensitivity of microfilaria testing as the prevalence of infection (and thus the number of circulating microfilaria overall and per infected person) decreases [7]. In child populations, smaller, lower-intensity microfoci are essentially invisible, due to the small size of the target population and the very low prevalence of microfilaremia in the background child population. In this model, microfilaria testing can identify large and intense microfoci, but even the best protocols only identified one of five microfoci when they were small and low-intensity, and all required testing of >5% of the total population. Combined with the need to sample persons in most areas between 10 pm and 2 am, microfilaria testing is unlikely to be a practical solution for monitoring the success of an elimination program. Using ICT identifies more, less intense, smaller endemic foci of infection while testing fewer persons.

We additionally show the challenges of cluster sampling, as compared with simple random sampling, in detecting microfoci. The primary advantages in cluster sampling are logistical, as fewer areas need to be visited during a survey than would be required for simple random sampling. However, even in settings of large, high-intensity microfoci, a maximum of only 40% of microfoci were identified in this model. In these protocols, relatively few persons are tested overall due to the low proportion of persons tested during primary sampling and the follow-up of only 20 persons around each positive. When considering small, low-intensity microfoci, a maximum of one of five microfoci were detected by cluster sampling, at a total cost of testing approximately 3% of the population. Using the ostensibly more sensitive Wb123 test does not yield a meaningful improvement on this metric, although it does improve the predictive value positive: ICT testing of adults or WCBA is clearly the most efficient way of detecting small, low-intensity microfoci regardless of whether cluster sampling or simple random sampling is used. Importantly, TAS-like sampling performed poorly in this model regardless of diagnostic test type used, microfocus size, or microfocus intensity; too few areas were covered during sampling to identify more than one of five microfoci in each simulation.

Microfoci, and not individually dispersed infections, pose the greatest risk for recrudescence of LF. However, the uncertainty surrounding what type of microfoci will spread without further intervention has been a stumbling block in setting LF elimination program targets. Conducting studies to determine which microfoci will spread is unethical: one cannot identify infected persons and deny treatment to evaluate the potential for infection propagation. Results from this model allow us to consider changing our approach to PTS entirely. Current methods focus on measuring average antigen prevalence, a metric that becomes less relevant as evaluation unit size and focal endemicity increase. Instead of targeting maximum tolerable average antigen prevalence for PTS, a decision about the maximum tolerable size and intensity of residual microfoci, and the confidence we desire in their absence in a post-MDA setting, could be considered. For example, we may wish to be 80% confident in the absence of residual microfoci >2 km in diameter, with intensities of infection ≥3 times the overall antigen prevalence in the population. We may choose to use ICT, and know that we want to test adult outpatients to approximate SRS. Under this framework, different simulations could be examined to determine which provided at least 80% confidence in the absence of such microfoci.

There are several limitations to this model. First, while we tried to design a conservative landscape that captured plausible post-MDA settings, there are currently few data to inform the true post-MDA situation with regard to residual infections or infection markers—particularly Wb123, for which the meaning of a positive test remains unclear. Because Wb123 was simply simulated as a more sensitive test than microfilaremia or ICT, it may be appropriate to consider the results for this test from that perspective, rather than as representing the actual performance of Wb123. Related to this, a minority of simulations did not yield detectable microfoci; that is, due to the low prevalence of background infections, particularly for children being tested for less-prevalent markers such as microfilaremia, even larger or more intense microfoci rarely had statistically significantly more infection-marker-positive persons than background. Thus, in these simulations, identification of microfoci was more a function of chance than of a true difference in infection marker prevalence. While this is important in terms of understanding model results, it is equally important in considering where the limits of detection lie with different markers. Notably, as mentioned above, it is unclear what comprises a microfocus that would spread without further intervention, and thus we cannot separate residual foci of infection into ‘important’ and ‘less important’. Beyond this, risk of LF is not homogeneously distributed across an area; however, precise prediction of risk from related factors is not available and because of this, we chose to treat all areas as though they were at equal risk. Improved determination of risk factors for infection ‘hotspots’ could facilitate better identification of priority areas for PTS, although this is unlikely to occur in time for most countries stopping MDA.

While this model was designed with LF in mind, it can also be applied to surveillance for other low-prevalence diseases, particularly those for which clinical signs cannot be used to estimate concurrent infection prevalence. Hepatitis B virus (HBV) is one such infection for which this might prove useful. Similar to LF, the signs and symptoms of HBV are not overt until many years after infection, and the elimination target involves reducing antigenemia to <2% in 5-year-old children (with the eventual goal of <1% antigenemia in the general population) [35]. Other diseases with nonspecific symptoms, such as malaria, could also benefit from similar modeling.

While this is a simulation, the parameters—particularly for small, non-intense microfoci—are not extraordinary. Large microfoci may be more easily detected during TAS or sentinel/spot-check sampling, although there is no guidance about follow-up testing or actions when persons are identified as positive. Data from this model provide two critical pieces of information: first, to be confident that microfoci, if they existed, would be detected, we need to carry out more robust surveillance than our current TAS requires. Second, microfilaremia testing is unlikely to be useful for PTS if confirmation of elimination is the goal.

To summarize, we show here that our current efforts at post-treatment surveillance will not suffice to detect small, low-intensity microfoci that may remain after cessation of MDA for LF. The use of more sensitive tests and more thorough testing methods are obligatory if we are to have confidence in long-term elimination of LF. Determining both practical and useful methods of surveillance may require some creativity, and perhaps graded efforts over time which help identify areas of interest during the first year, followed by much smaller continued surveys in subsequent years. High-intensity sampling of TAS-eligible areas would provide important data to improve this type of modeling.

## Supporting information

S1 Details Details of the microsimulation model. (DOCX)

S1 Fig Density plots of ten 60 km x 60 km simulation areas used for modeling. (TIF)

S1 Table Inputs and outputs of simulation models with five microfoci per simulation area (n = 3,402). Variables as follows: Test: Test type used. Samp: Sampling methodology. PP: Population proportion sampled. Ages: ages sampled during primary sampling. Radius: microfocus radius. Int: microfocus intensity. TBS: Number tested in trigger-based sampling (all ages). TH: Threshold (number of positives required during trigger-based sampling for program manager to believe they have identified a microfocus). Pos/Test: Proportion of persons tested who are positive. Pos/PopPos: Proportion of all positive persons in the population who are identified in testing. PVP (Median): Median proportion of all suspected microfoci that were correctly identified as microfoci. PVP (LCL): 2.5^th^ percentile for PVP. PVP (UCL): 97.5^th^ percentile for PVP. Test/Pop (Median): Median proportion of total population tested. Test/Pop (UCL): 2.5^th^ percentile for Test/Pop. Test/Pop (LCL): 97.5^th^ percentile for Test/Pop. Sensitivity (Median): Median proportion of all microfoci found through the sampling protocol. Sensitivity (LCL): 2.5^th^ percentile for sensitivity. Sensitivity (UCL): 97.5^th^ percentile for sensitivity.
(CSV)

S1 File R code used to generate and run microsimulation model. (ZIP)
