# Supplementary material for: Detecting infection hotspots: Modeling the surveillance challenge for elimination of lymphatic filariasis
Source: PLoS Negl Trop Dis. 2017 May 19;11(5):e0005610. doi: 10.1371/journal.pntd.0005610 (PMC5453617; doi:10.1371/journal.pntd.0005610)
Supplement: S1 Details — (DOCX) [file pntd.0005610.s001.docx]

***Supplemental material: Simulation details***

All simulations were run in R versions 3.1 and 3.2 [1].

First, village centroids were placed with complete spatial randomness in the region [2]. Based on these locations, the region was tessellated using a Delaunay triangulation [3, 4] to create village boundaries and converted to spatial polygons with the sp package [5]. The population size of a village was then randomly determined based on its area, where the probability of an individual being in a village was equal to the area of village divided by the total area. Next, the number of households in each village was determined by dividing the village population by the average number of persons per household in the village and rounding to the nearest integer. The average number of persons per household was randomly generated from a truncated normal distribution with a mean of six, standard deviation of one, lower bound of four, and upper bound of eight [6].

Household locations were then randomly created by first simulating an angle from 0 to 2π from a uniform distribution. The distance from the centroid to the village boundary was calculated and a distance from the centroid was generated from a Beta distribution with $\alpha=1$ and $\beta=3$. This allows for the possibility for households to be located anywhere in the region and for a higher density of households to be located near the centroid.

Each person’s sex was determined from a uniform distribution with $p=0.5$ and ages were generated based on the population pyramid (Table 1). First, we placed children randomly in each household, with equal probability, though we required at least one adult to be in each household. The adults were randomly placed in the remaining slots, again with equal probability. With the region complete, microfoci were placed on the map. Microfoci had two requirements: (1) an existing household must serve as a microfocus centroid to insure that at least one household would be included in each microfoci, and (2) microfoci must be at least 1 km apart [7].

Once the required set of microfoci were established, then the disease status of each person was determined. Probabilities of being positive were broken down by age group (Table 1). We assumed that all microfilaremia (mf)-positive persons were also positive by ICT, and all positives by ICT were also positive by WB123. Based on the correlations between tests observed in a Haitian sample of children [8], this assumption is reasonable. Hence, mf positives are a subset of ICT positives which are a subset of WB123 positives. The proportions for WB123 were created first, as this test generates the most positives. Next, ICT test results were generated based on a random sample of those positive for WB123. Finally, mf positives were determined based on a random sample of those positive by ICT.

Next, random samples were taken from each of the subpopulations. From each subpopulation, we took a simple random sample and a multistage cluster sample. The cluster sample was taken from 30 randomly-selected communities. The subpopulation in each of the 30 communities was scaled downward based on the following formula

$S_{j}=n_{j}-\left( \frac{n_{j}}{\sum_{j} n_{j}} \right)\left( \sum_{j} n_{j}-N \right)$,

where $S_{j}$is the number to be sampled from village $j$’s subpopulation, $n_{j}$is the total subpopulation in village $j$, and $N$is the total number to be sampled. Within each village, households were placed in random order. All persons within a selected household are sampled. Hence, the first $h_{j}$households that come the closest in absolute value to $S_{j}$were sampled. This may result in a total sample size slightly smaller or larger than the desired sample size, but should, on the average, give the desired sample size.

The number of positive persons (initial cases) sampled for each subsample and test combination were calculated. For example, starting with a given population sampled and a given test, for each case identified during primary sampling, the program manager activates trigger-based sampling around that case. Euclidian distances between all primary cases and the entire population were determined and the nearest $r$ persons to each primary case were identified [9, 10]. As described above, we chose three values for $r$ (20, 100, and 500). If the threshold is met for the identification of positives among persons tested, a polygon with a buffer of 0.5 km is drawn around each of the trigger-sampled persons and the primary case that met the threshold [11-13]. This polygon is considered a ‘suspected microfocus.’ For ease in the simulation process, if all trigger-sampled persons were from the same household, a circle with a 10 meter radius was drawn around the household. If all were from two households, then an ellipse was drawn around those households with the sum of distances to each point on the curve equal to the distance between the foci plus 10 meters. If any of these polygons intersect, those polygons are joined together and are considered to be a single suspected microfocus [13]. At the end of this process, these and the remaining, unique suspected microfoci are counted and recorded. If any positive individual in the microfocus fell inside of a true microfocus placed on the map, that individual was considered a ‘true positive’, while all suspected microfoci that did not contain any individuals identified as positive who fell inside of a true microfocus were considered ‘false positives.’ Finally, the total number of persons tested and total number of cases detected are recorded as well as whether or not all microfoci were found in the simulation.

The process described above was performed 1,000 times for all combinations of parameters. However, due to the computationally-intense process of creating and placing households, we created ten regions and ran 100 simulations on each region (Supplementary Figures 1a-1j).

Data were organized and summarized in R [14, 15] and analyzed in R and Excel.

**Supplementary Figure 1.** Density plots of ten 60 km x 60 km simulation areas used for modeling.

**Supplementary Table 1.** Inputs and outputs of simulation models with five microfoci per simulation area (n=3,402). Variables as follows: Test: Test type used. Samp: Sampling methodology. PP: Population proportion sampled. Ages: ages sampled during primary sampling. Radius: microfocus radius. Int: microfocus intensity. TBS: Number tested in trigger-based sampling (all ages). TH: Threshold (number of positives required during trigger-based sampling for program manager to believe they have identified a microfocus). Pos/Test: Proportion of persons tested who are positive. Pos/PopPos: Proportion of all positive persons in the population who are identified in testing. PVP (Median): Median proportion of all suspected microfoci that were correctly identified as microfoci. PVP (LCL): 2.5^th^ percentile for PVP. PVP (UCL): 97.5^th^ percentile for PVP. Test/Pop (Median): Median proportion of total population tested. Test/Pop (UCL): 2.5^th^ percentile for Test/Pop. Test/Pop (LCL): 97.5^th^ percentile for Test/Pop. Sensitivity (Median): Median proportion of all microfoci found through the sampling protocol. Sensitivity (LCL): 2.5^th^ percentile for sensitivity. Sensitivity (UCL): 97.5^th^ percentile for sensitivity.

**Supplementary File 1.** R code used to generate and run microsimulation model.

**References**

1. R Core Team. R: A language and environment for statistical computing. Vienna, Austria: R Foundation for Statistical Computing; 2015.

2. Rowlingson B, Diggle P. splancs: Spatial and Space-Time Point Pattern Analysis. R package version 2.01-37 ed2015.

3. Lee DT, Schacter BJ. Two algorithms for constructing a Delaunay triangulation. International Journal of Computer and Information Sciences. 1980;9(3):219-42.

4. Turner R. deldir: Delaunay Triangulation and Dirichlet (Voronoi) Tessellation. R package version 0.1-9 ed2015.

5. Bivand RS, Pebesma E, Gomez-Rubio V. Applied spatial data analysis with R. 2nd ed. New York: Springer; 2013.

6. Trautmann H, Steuer D, Mersmann O, Bornkamp B. truncnorm: Truncated normal distribution. R package version 1.0-7. ed2014.

7. Nychka D, Furrer R, Paige J, Sain S. fields: Tools for Spatial Data. R package version 8.3-5 ed2015.

8. Hamlin KL, Moss DM, Priest JW, Roberts J, Kubofcik J, Gass K, et al. Longitudinal monitoring of the development of antifilarial antibodies and acquisition of Wuchereria bancrofti in a highly endemic area of Haiti. PLoS neglected tropical diseases. 2012;6(12):e1941. doi: 10.1371/journal.pntd.0001941. PubMed PMID: 23236534; PubMed Central PMCID: PMC3516578.

9. Arya S, Mount DM, Netanyahu NS, Silverman R, Wu AY. An optimal algorithm for approximate nearest neighbor searching. Journal of the ACM. 1998;45(6):891-923.

10. Beygelzimer A, Kakadet S, Langford J, Arya S, Mount D, Li S. FNN: Fast Nearest Neighbor Search Algorithms and Applications. 2013.

11. Eddy WF. A new convex hull algorithm for planar sets. ACM Transactions on Mathematical Software. 1977;3(4):398-403.

12. Eddy WF. Algorithm 523: CONVEX, A New Convex Hull Algorithm for Planar Sets [Z]. ACM Transactions on Mathematical Software. 1977;3(4):411-2.

13. Bivand R, Rundel C. rgeos: Interface to Geometry Engine - Open Source (GEOS). R package version 0.3-15 ed2015.

14. Wickham H. Reshaping Data with the reshape Package. Journal of Statistical Software. 2007;21(12):1-20.

15. Wickham H. The Split-Apply-Combine Strategy for Data Analysis. Journal of Statistical Software. 2011;40(1):1-29.
