# Supplementary figures and images for: Detecting infection hotspots: Modeling the surveillance challenge for elimination of lymphatic filariasis
Source: PLoS Negl Trop Dis. 2017 May 19;11(5):e0005610. doi: 10.1371/journal.pntd.0005610 (PMC5453617; doi:10.1371/journal.pntd.0005610)

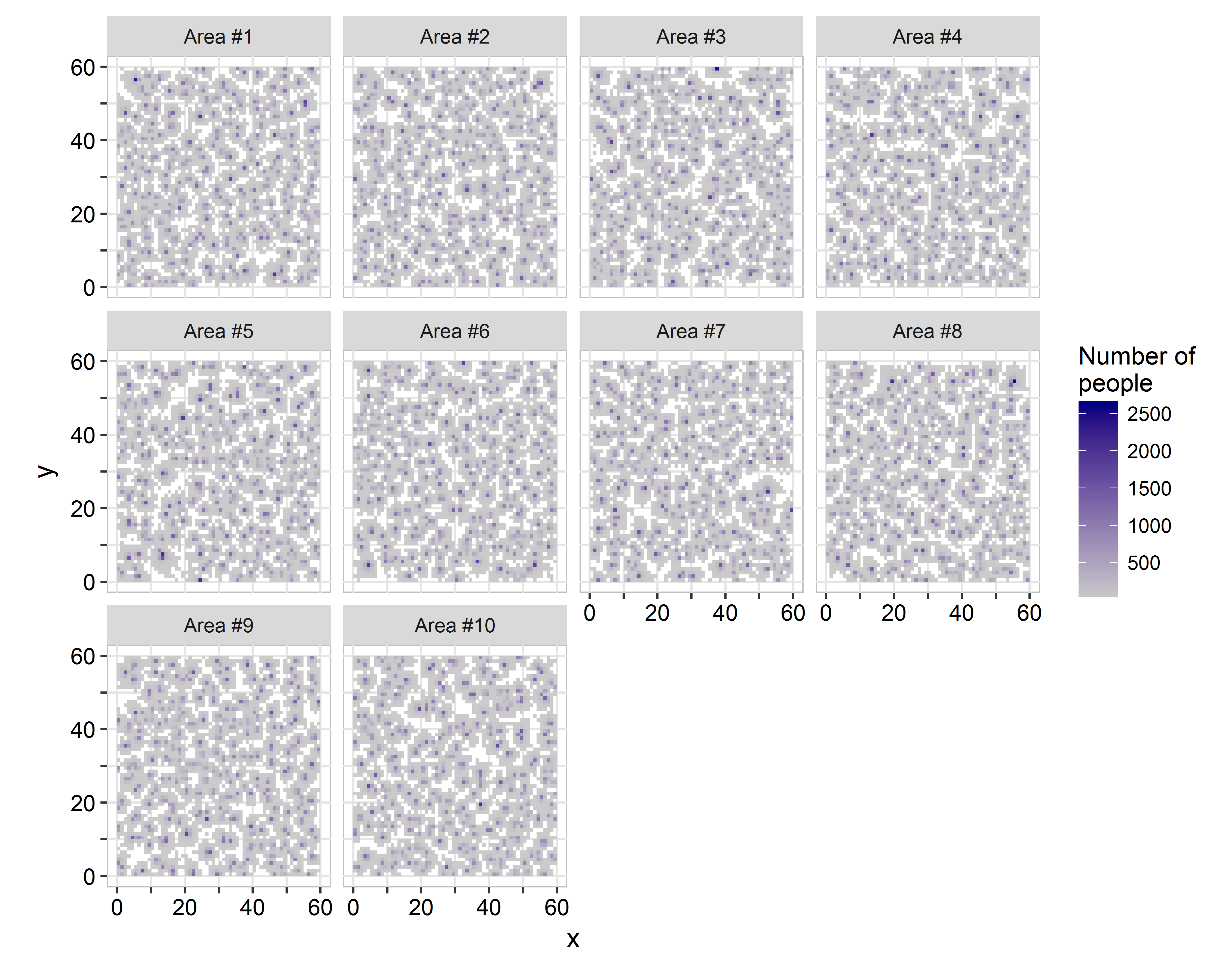

Supplement: S1 Fig — (TIF) [file pntd.0005610.s002.tif]
